# *cis*-Bis(2,2′-bipyridine-κ^2^ *N*,*N*′)carbonyl­chloridoruthenium(II) hexa­fluorido­phosphate

**DOI:** 10.1107/S1600536812048246

**Published:** 2012-12-05

**Authors:** Tsugiko Takase, Bisa Mun, Dai Oyama

**Affiliations:** a Center for Practical and Project-Based Learning, Cluster of Science and Technology, Fukushima University, 1 Kanayagawa, Fukushima 960-1296, Japan; b Department of Materials Science, Graduate School of Science and Technology, Fukushima University, 1 Kanayagawa, Fukushima 960-1296, Japan; c Department of Industrial Systems Engineering, Cluster of Science and Technology, Fukushima University, 1 Kanayagawa, Fukushima 960-1296, Japan

## Abstract

In the title compound, [RuCl(C_10_H_8_N_2_)_2_(CO)]PF_6_, the Ru^II^ atom is coordinated in a distorted octa­hedral geometry by four N atoms of the bipyridine ligands, a carbonyl C atom and a chloride ion. The carbonyl and chloride ligands in the cation adopt a mutually *cis* arrangement and these are disordered over two sets of sites with site occupancies of 0.721 (6) and 0.279 (6). The Ru—N bond length [2.117 (2) Å] *trans* to the carbonyl ligand is slightly longer than the average of the other Ru—N bond lengths (2.08 Å), which can be explained by the expected *trans* influence of the carbonyl group. In the crystal, weak C—H⋯F inter­actions are observed between the complex cation and the PF_6_^−^ anion, leading to the formation of a three-dimensional supramolecular structure. The crystal studied was an inversion twin with twin fractions of 0.78 (4) and 0.22 (4).

## Related literature

For details of the synthesis, see: Oyama *et al.* (2012 ▶). For a related structure, see: Clear *et al.* (1980 ▶). For general background to catalytic reactions using [Ru(bpy)_2_(CO)Cl]^+^, see: Ishida *et al.* (1986 ▶); Lehn & Ziessel (1990 ▶).
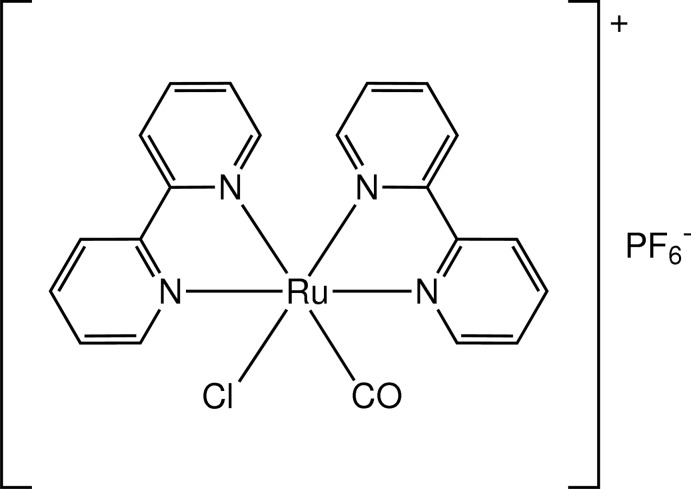


## Experimental

### 

#### Crystal data

[RuCl(C_10_H_8_N_2_)_2_(CO)]·PF_6_
*M* *_r_* = 621.87
Orthorhombic, 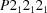

*a* = 10.882 (5) Å
*b* = 12.063 (5) Å
*c* = 17.410 (7) Å
*V* = 2285.2 (17) Å^3^
*Z* = 4
Mo *K*α radiation
μ = 0.95 mm^−1^
*T* = 93 K
0.20 × 0.10 × 0.02 mm

#### Data collection

Rigaku Saturn diffractometer
Absorption correction: multi-scan (*REQAB*; Jacobson, 1998 ▶) *T*_min_ = 0.897, *T*_max_ = 0.981
22875 measured reflections
5177 independent reflections
4689 reflections with *F* ^2^ > 2σ(*F* ^2^)
*R*_int_ = 0.045

#### Refinement

*R*[*F* ^2^ > 2σ(*F* ^2^)] = 0.032
*wR*(*F* ^2^) = 0.062
*S* = 1.08
5177 reflections
330 parameters
H-atom parameters constrained
Δρ_max_ = 1.37 e Å^−3^
Δρ_min_ = −1.28 e Å^−3^
Absolute structure: Flack (1983 ▶), 2249 Friedel pairs
Flack parameter: 0.22 (4)

### 

Data collection: *CrystalClear-SM* (Rigaku, 2009 ▶); cell refinement: *CrystalClear-SM*; data reduction: *CrystalClear-SM*; program(s) used to solve structure: *SIR97* (Altomare *et al.*, 1999 ▶); program(s) used to refine structure: *SHELXL97* (Sheldrick, 2008 ▶); molecular graphics: *ORTEP-3 for Windows* (Farrugia, 2012 ▶); software used to prepare material for publication: *CrystalStructure* (Rigaku, 2006 ▶).

## Supplementary Material

Crystal structure: contains datablock(s) global, I. DOI: 10.1107/S1600536812048246/is5220sup1.cif

Structure factors: contains datablock(s) I. DOI: 10.1107/S1600536812048246/is5220Isup2.hkl

Additional supplementary materials:  crystallographic information; 3D view; checkCIF report

## Figures and Tables

**Table 1:** Selected bond lengths (Å)

Ru1—Cl1	2.3521 (17)
Ru1—N1	2.086 (2)
Ru1—N2	2.070 (2)
Ru1—N3	2.070 (2)
Ru1—N4	2.117 (2)
Ru1—C21	1.890 (8)

**Table 2:** Hydrogen-bond geometry (Å, °)

*D*—H⋯*A*	*D*—H	H⋯*A*	*D*⋯*A*	*D*—H⋯*A*
C3—H3⋯F2^i^	0.95	2.46	3.166 (4)	131
C4—H4⋯F1^ii^	0.95	2.41	3.257 (4)	148
C7—H5⋯F1^ii^	0.95	2.54	3.431 (4)	156
C8—H6⋯F2^iii^	0.95	2.50	3.265 (4)	138
C13—H11⋯F5^iv^	0.95	2.39	3.331 (4)	168

Symmetry codes: (i) 
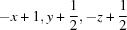
; (ii) 
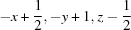
; (iii) 
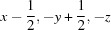
; (iv) 
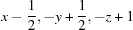
.

**Fig. 1.**
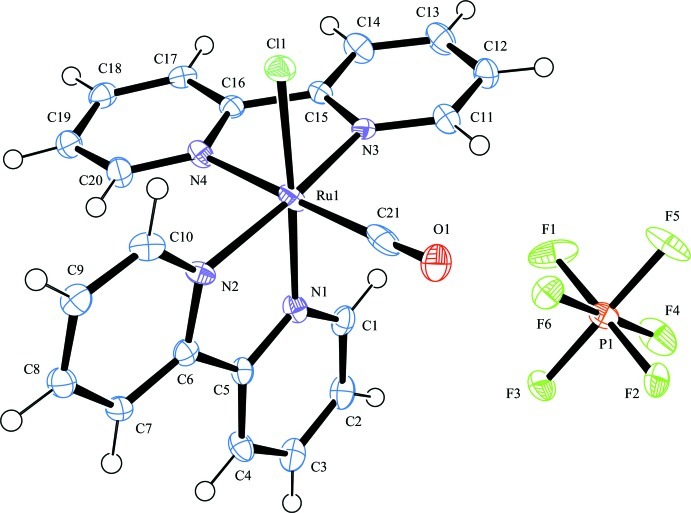
The molecular structure of the title compound, with atom labels and 50% probability displacement ellipsoids for non-H atoms. Only major component of the disordered CO and Cl ligands is shown.

## supplementary crystallographic information

### Comment

Ruthenium(II) complexes containing both carbonyl and polypyridyl-based supporting ligands have been studied as catalysts for the reduction of carbon dioxide and in the water-gas shift reaction (Ishida *et al.*, 1986; Lehn & Ziessel, 1990). Of the series complexes, [Ru(bpy)_2_(CO)Cl]^+^ (bpy = 2,2'-bipyridine) has been used not only as catalysts but also as a precursor to a family of [Ru(bpy)_2_(CO)*L*]*^n^*^+^-type complexes (*L* = monodentate ligand). We have used the complex as the starting point for the preparation of more complex functional systems, and we report here the crystal structure of its hexafluoridophosphate salt.

The Ru^II^ atom has a distorted octahedral geometry, with four N atoms of the bidentate bipyridine ligands, a carbonyl carbon, and a chloride ion completing the first coordination sphere. The CO and Cl ligands in the cation are mutually *cis* arrangement (Fig. 1) and these are disordered over two sets of sites with site occupancies of 0.721 (6) and 0.279 (6). The Ru—N length *trans* to the CO ligand [2.117 (2) Å] is slightly longer than the average of other Ru—N lengths (2.08 Å) (Table 1). This can be explained by the expected *trans* influence of the CO group. In the crystal, the complex cation and the PF_6_^-^ anion are linked *via* a number of weak C—H···F interactions, leading to the formation of a three-dimensional supramolecular structure. The crystal studied was an inversion twin with twin fractions of 0.78 (4) and 0.22 (4). The bond parameters of the complex are closely comparable to those of the reported ClO_4_^-^ salt, although the corresponding ClO_4_^-^ salt was refined using anisotropic temperature factors for Ru and Cl only (Clear *et al.*, 1980).

### Experimental

The title compound was prepared according to a literature procedure (Oyama *et al.*, 2012). X-ray quality crystals were grown by the diffusion of diethyl ether into an acetone solution of the complex over a week.

### Refinement

Aromatic H atoms were fixed at C—H distances of 0.95 Å and refined as riding, with *U*_iso_(H) = 1.2*U*_eq_(C). The C and O atoms in the CO group and the Cl atom are disordered over two sets of sites, occupancies refining to 0.721 (6) and complement. Both the highest residual electron density peak and the deepest hole are located within 1 Å from atom Ru1. The Hooft y parameter was 0.228 (15).

### Crystal data

**Table:**

[RuCl(C_10_H_8_N_2_)_2_(CO)]·PF_6_	*F*(000) = 1232.00
*M**_r_* = 621.87	*D*_x_ = 1.807 Mg m^−^^3^
Orthorhombic, *P*2_1_2_1_2_1_	Mo *K*α radiation, λ = 0.71075 Å
Hall symbol: P 2ac 2ab	Cell parameters from 6659 reflections
*a* = 10.882 (5) Å	θ = 3.4–27.4°
*b* = 12.063 (5) Å	µ = 0.95 mm^−^^1^
*c* = 17.410 (7) Å	*T* = 93 K
*V* = 2285.2 (17) Å^3^	Block, orange
*Z* = 4	0.20 × 0.10 × 0.02 mm

### Data collection

**Table:**

Rigaku Saturn diffractometer	4689 reflections with *F*^2^ > 2σ(*F*^2^)
Detector resolution: 7.31 pixels mm^-1^	*R*_int_ = 0.045
ω scans	θ_max_ = 27.4°
Absorption correction: multi-scan (*REQAB*; Jacobson, 1998)	*h* = −14→14
*T*_min_ = 0.897, *T*_max_ = 0.981	*k* = −15→15
22875 measured reflections	*l* = −22→22
5177 independent reflections	

### Refinement

**Table:**

Refinement on *F*^2^	*w* = 1/[σ^2^(*F*_o_^2^) + (0.0116*P*)^2^ + 2.2808*P*] where *P* = (*F*_o_^2^ + 2*F*_c_^2^)/3
*R*[*F*^2^ > 2σ(*F*^2^)] = 0.032	(Δ/σ)_max_ = 0.001
*wR*(*F*^2^) = 0.062	Δρ_max_ = 1.37 e Å^−^^3^
*S* = 1.08	Δρ_min_ = −1.28 e Å^−^^3^
5177 reflections	Absolute structure: Flack (1983), 2249 Friedel pairs
330 parameters	Flack parameter: 0.22 (4)
H-atom parameters constrained	

### Special details

**Table:**

Refinement. Refinement was performed using all reflections. The weighted *R*-factor (*wR*) and goodness of fit (*S*) are based on *F*^2^. *R*-factor (gt) are based on *F*. The threshold expression of *F*^2^ > 2.0 σ(*F*^2^) is used only for calculating *R*-factor (gt).

### Fractional atomic coordinates and isotropic or equivalent isotropic displacement parameters (Å^2^)

**Table:**

	*x*	*y*	*z*	*U*_iso_*/*U*_eq_	Occ. (<1)
Ru1	0.02797 (3)	0.23729 (2)	0.135509 (15)	0.01786 (6)	
Cl1	−0.09514 (14)	0.07702 (15)	0.13630 (11)	0.0212 (5)	0.721 (6)
Cl2	0.1924 (6)	0.1234 (4)	0.1216 (3)	0.0179 (16)*	0.279 (6)
P1	0.43909 (9)	0.30250 (7)	0.35040 (5)	0.0207 (2)	
F1	0.3130 (2)	0.35010 (18)	0.38378 (14)	0.0404 (6)	
F2	0.56516 (17)	0.25391 (18)	0.31614 (11)	0.0265 (4)	
F3	0.4530 (2)	0.41078 (15)	0.29742 (11)	0.0267 (4)	
F4	0.5138 (2)	0.36277 (16)	0.41762 (12)	0.0366 (6)	
F5	0.4261 (2)	0.19318 (16)	0.40319 (12)	0.0366 (6)	
F6	0.36435 (17)	0.24099 (19)	0.28323 (11)	0.0254 (4)	
O1	0.2506 (4)	0.0882 (3)	0.1258 (2)	0.0295 (10)	0.721 (6)
O2	−0.1251 (10)	0.0222 (11)	0.1282 (6)	0.027 (2)*	0.279 (6)
N1	0.1347 (2)	0.3805 (2)	0.12773 (18)	0.0172 (6)	
N2	0.0354 (2)	0.2586 (2)	0.01763 (13)	0.0158 (5)	
N3	0.0041 (2)	0.2422 (2)	0.25340 (14)	0.0159 (5)	
N4	−0.1287 (2)	0.3405 (2)	0.14489 (17)	0.0168 (6)	
C1	0.1837 (3)	0.4363 (3)	0.1868 (2)	0.0207 (7)	
C2	0.2568 (3)	0.5285 (3)	0.1763 (2)	0.0226 (8)	
C3	0.2804 (3)	0.5650 (2)	0.1025 (2)	0.0230 (8)	
C4	0.2297 (3)	0.5082 (2)	0.0408 (2)	0.0200 (7)	
C5	0.1558 (3)	0.4162 (2)	0.05464 (19)	0.0153 (7)	
C6	0.0964 (3)	0.3508 (2)	−0.00646 (19)	0.0163 (7)	
C7	0.0989 (3)	0.3809 (2)	−0.0836 (2)	0.0183 (7)	
C8	0.0351 (3)	0.3163 (2)	−0.1360 (2)	0.0220 (6)	
C9	−0.0250 (3)	0.2223 (2)	−0.11146 (18)	0.0203 (6)	
C10	−0.0236 (3)	0.1960 (2)	−0.03456 (19)	0.0203 (7)	
C11	0.0752 (3)	0.1916 (2)	0.3056 (2)	0.0255 (8)	
C12	0.0524 (3)	0.1979 (2)	0.3838 (2)	0.0251 (8)	
C13	−0.0466 (3)	0.2590 (3)	0.40917 (18)	0.0266 (7)	
C14	−0.1208 (3)	0.3123 (2)	0.3563 (2)	0.0240 (7)	
C15	−0.0943 (3)	0.3029 (2)	0.27831 (19)	0.0159 (7)	
C16	−0.1674 (3)	0.3575 (2)	0.2178 (2)	0.0159 (7)	
C17	−0.2693 (3)	0.4242 (2)	0.2327 (2)	0.0186 (7)	
C18	−0.3292 (3)	0.4755 (2)	0.1729 (2)	0.0215 (8)	
C19	−0.2875 (3)	0.4598 (3)	0.0984 (2)	0.0238 (8)	
C20	−0.1878 (3)	0.3906 (3)	0.0875 (2)	0.0218 (8)	
C21	0.1713 (8)	0.1488 (6)	0.1328 (5)	0.0264 (19)	0.721 (6)
C22	−0.080 (2)	0.1111 (17)	0.1316 (15)	0.033 (6)*	0.279 (6)
H1	0.1674	0.4116	0.2376	0.025*	
H2	0.2905	0.5664	0.2193	0.027*	
H3	0.3307	0.6282	0.0940	0.028*	
H4	0.2453	0.5319	−0.0103	0.024*	
H5	0.1434	0.4444	−0.1000	0.022*	
H6	0.0328	0.3368	−0.1887	0.026*	
H7	−0.0668	0.1761	−0.1471	0.024*	
H8	−0.0658	0.1315	−0.0178	0.024*	
H9	0.1439	0.1499	0.2884	0.031*	
H10	0.1042	0.1606	0.4193	0.030*	
H11	−0.0637	0.2646	0.4626	0.032*	
H12	−0.1893	0.3548	0.3730	0.029*	
H13	−0.2971	0.4342	0.2840	0.022*	
H14	−0.3986	0.5212	0.1826	0.026*	
H15	−0.3262	0.4956	0.0562	0.029*	
H16	−0.1601	0.3781	0.0365	0.026*	

### Atomic displacement parameters (Å^2^)

**Table:**

	*U*^11^	*U*^22^	*U*^33^	*U*^12^	*U*^13^	*U*^23^
Ru1	0.02530 (13)	0.01498 (11)	0.01331 (11)	0.00333 (12)	0.00509 (12)	0.00234 (11)
Cl1	0.0230 (7)	0.0190 (11)	0.0215 (7)	−0.0062 (6)	−0.0010 (6)	0.0016 (7)
P1	0.0266 (5)	0.0181 (4)	0.0175 (5)	−0.0013 (3)	0.0036 (3)	−0.0010 (3)
F1	0.0323 (13)	0.0377 (13)	0.0512 (18)	−0.0069 (10)	0.0251 (11)	−0.0185 (11)
F2	0.0229 (10)	0.0255 (10)	0.0311 (10)	0.0082 (10)	−0.0030 (8)	0.0033 (10)
F3	0.0298 (13)	0.0196 (9)	0.0306 (12)	0.0037 (9)	0.0006 (9)	0.0086 (8)
F4	0.0600 (17)	0.0284 (10)	0.0215 (11)	−0.0152 (11)	−0.0104 (11)	−0.0022 (8)
F5	0.0713 (19)	0.0218 (10)	0.0167 (11)	−0.0173 (11)	0.0028 (11)	0.0042 (8)
F6	0.0251 (10)	0.0268 (11)	0.0244 (10)	−0.0022 (10)	−0.0034 (8)	−0.0066 (9)
O1	0.024 (2)	0.026 (2)	0.039 (2)	0.0095 (19)	−0.0004 (19)	0.0067 (18)
N1	0.0216 (15)	0.0180 (13)	0.0121 (15)	0.0040 (11)	0.0011 (13)	−0.0017 (12)
N2	0.0201 (14)	0.0134 (12)	0.0140 (12)	−0.0018 (14)	0.0044 (11)	−0.0008 (10)
N3	0.0141 (14)	0.0190 (13)	0.0147 (12)	−0.0027 (11)	0.0012 (9)	0.0025 (11)
N4	0.0239 (15)	0.0151 (13)	0.0116 (15)	0.0003 (11)	−0.0002 (13)	−0.0018 (11)
C1	0.0169 (19)	0.0297 (19)	0.0153 (18)	0.0050 (15)	−0.0023 (14)	−0.0040 (15)
C2	0.026 (2)	0.020 (2)	0.022 (2)	0.0020 (16)	−0.0089 (16)	−0.0070 (15)
C3	0.024 (2)	0.0150 (17)	0.030 (2)	−0.0003 (15)	−0.0088 (16)	−0.0019 (15)
C4	0.028 (2)	0.0141 (17)	0.0185 (18)	−0.0008 (15)	−0.0035 (15)	0.0021 (13)
C5	0.0173 (18)	0.0134 (16)	0.0152 (17)	0.0017 (13)	−0.0033 (14)	−0.0012 (13)
C6	0.0152 (17)	0.0149 (16)	0.0188 (18)	0.0034 (13)	0.0006 (13)	−0.0007 (13)
C7	0.0173 (18)	0.0190 (17)	0.0186 (18)	−0.0043 (14)	−0.0007 (14)	0.0027 (13)
C8	0.0235 (17)	0.0278 (15)	0.0146 (16)	−0.0045 (15)	−0.0016 (19)	−0.0008 (14)
C9	0.0211 (16)	0.0213 (15)	0.0186 (16)	−0.0009 (17)	−0.0008 (14)	−0.0059 (12)
C10	0.0223 (17)	0.0141 (14)	0.0243 (18)	−0.0012 (16)	0.0053 (16)	−0.0044 (12)
C11	0.026 (2)	0.0279 (19)	0.0225 (19)	0.0012 (16)	−0.0009 (16)	0.0075 (15)
C12	0.026 (2)	0.0299 (18)	0.0191 (19)	−0.0031 (15)	−0.0068 (14)	0.0050 (13)
C13	0.038 (2)	0.0280 (18)	0.0140 (14)	−0.0004 (19)	−0.0012 (14)	0.0008 (15)
C14	0.032 (2)	0.0218 (16)	0.0178 (18)	0.0012 (14)	0.0023 (17)	−0.0022 (15)
C15	0.0179 (17)	0.0160 (15)	0.0140 (16)	−0.0010 (13)	0.0012 (13)	0.0013 (12)
C16	0.0179 (18)	0.0130 (16)	0.0167 (17)	−0.0040 (13)	−0.0010 (13)	−0.0008 (12)
C17	0.0165 (17)	0.0177 (17)	0.0216 (19)	−0.0022 (13)	0.0038 (14)	−0.0015 (14)
C18	0.0165 (19)	0.0169 (17)	0.031 (2)	0.0016 (15)	0.0014 (16)	0.0010 (15)
C19	0.021 (2)	0.0210 (18)	0.029 (2)	0.0009 (15)	−0.0025 (17)	0.0074 (16)
C20	0.023 (2)	0.0237 (19)	0.0193 (19)	0.0031 (15)	−0.0012 (15)	0.0017 (15)
C21	0.042 (5)	0.015 (3)	0.022 (3)	−0.008 (3)	−0.001 (3)	0.006 (3)

### Geometric parameters (Å, º)

**Table:**

Ru1—Cl1	2.3521 (17)	C6—C7	1.391 (4)
Ru1—Cl2	2.269 (6)	C7—C8	1.387 (4)
Ru1—N1	2.086 (2)	C8—C9	1.377 (4)
Ru1—N2	2.070 (2)	C9—C10	1.376 (4)
Ru1—N3	2.070 (2)	C11—C12	1.385 (5)
Ru1—N4	2.117 (2)	C12—C13	1.378 (5)
Ru1—C21	1.890 (8)	C13—C14	1.382 (4)
Ru1—C22	1.93 (2)	C14—C15	1.393 (4)
P1—F1	1.597 (2)	C15—C16	1.476 (4)
P1—F2	1.607 (2)	C16—C17	1.394 (4)
P1—F3	1.606 (2)	C17—C18	1.376 (5)
P1—F4	1.600 (2)	C18—C19	1.387 (5)
P1—F5	1.614 (2)	C19—C20	1.382 (5)
P1—F6	1.606 (2)	C1—H1	0.950
O1—C21	1.138 (9)	C2—H2	0.950
O2—C22	1.18 (2)	C3—H3	0.950
N1—C1	1.339 (4)	C4—H4	0.950
N1—C5	1.363 (4)	C7—H5	0.950
N2—C6	1.362 (4)	C8—H6	0.950
N2—C10	1.344 (4)	C9—H7	0.950
N3—C11	1.341 (4)	C10—H8	0.950
N3—C15	1.368 (4)	C11—H9	0.950
N4—C16	1.353 (4)	C12—H10	0.950
N4—C20	1.334 (4)	C13—H11	0.950
C1—C2	1.380 (5)	C14—H12	0.950
C2—C3	1.383 (5)	C17—H13	0.950
C3—C4	1.388 (5)	C18—H14	0.950
C4—C5	1.391 (4)	C19—H15	0.950
C5—C6	1.474 (4)	C20—H16	0.950
			
Cl1—Ru1—N1	176.52 (9)	C4—C5—C6	123.7 (3)
Cl1—Ru1—N2	97.47 (8)	N2—C6—C5	115.4 (2)
Cl1—Ru1—N3	86.91 (8)	N2—C6—C7	121.3 (2)
Cl1—Ru1—N4	91.41 (8)	C5—C6—C7	123.3 (2)
Cl1—Ru1—C21	90.3 (2)	C6—C7—C8	118.6 (3)
Cl2—Ru1—N1	93.18 (18)	C7—C8—C9	119.7 (3)
Cl2—Ru1—N2	86.45 (17)	C8—C9—C10	119.1 (3)
Cl2—Ru1—N3	102.85 (17)	N2—C10—C9	122.3 (2)
Cl2—Ru1—N4	177.80 (17)	N3—C11—C12	122.5 (3)
Cl2—Ru1—C22	90.0 (6)	C11—C12—C13	119.0 (3)
N1—Ru1—N2	79.12 (11)	C12—C13—C14	119.5 (3)
N1—Ru1—N3	96.35 (11)	C13—C14—C15	119.3 (3)
N1—Ru1—N4	88.06 (10)	N3—C15—C14	121.0 (2)
N1—Ru1—C21	90.4 (2)	N3—C15—C16	115.8 (2)
N1—Ru1—C22	173.1 (7)	C14—C15—C16	123.2 (3)
N2—Ru1—N3	169.95 (10)	N4—C16—C15	115.7 (2)
N2—Ru1—N4	92.01 (11)	N4—C16—C17	120.7 (3)
N2—Ru1—C21	90.8 (2)	C15—C16—C17	123.6 (3)
N2—Ru1—C22	95.0 (7)	C16—C17—C18	119.7 (3)
N3—Ru1—N4	78.80 (10)	C17—C18—C19	119.4 (3)
N3—Ru1—C21	98.3 (2)	C18—C19—C20	117.9 (3)
N3—Ru1—C22	88.9 (7)	N4—C20—C19	123.3 (3)
N4—Ru1—C21	176.5 (2)	Ru1—C21—O1	172.4 (7)
N4—Ru1—C22	88.6 (6)	Ru1—C22—O2	166.8 (18)
F1—P1—F2	179.39 (12)	N1—C1—H1	118.9
F1—P1—F3	89.87 (11)	C2—C1—H1	118.9
F1—P1—F4	90.40 (13)	C1—C2—H2	120.5
F1—P1—F5	90.64 (13)	C3—C2—H2	120.5
F1—P1—F6	89.76 (11)	C2—C3—H3	120.4
F2—P1—F3	90.16 (11)	C4—C3—H3	120.4
F2—P1—F4	90.20 (12)	C3—C4—H4	120.4
F2—P1—F5	89.32 (12)	C5—C4—H4	120.4
F2—P1—F6	89.63 (10)	C6—C7—H5	120.7
F3—P1—F4	90.14 (10)	C8—C7—H5	120.7
F3—P1—F5	179.47 (13)	C7—C8—H6	120.1
F3—P1—F6	90.31 (11)	C9—C8—H6	120.1
F4—P1—F5	89.96 (11)	C8—C9—H7	120.4
F4—P1—F6	179.52 (12)	C10—C9—H7	120.4
F5—P1—F6	89.58 (11)	N2—C10—H8	118.9
Ru1—N1—C1	126.0 (2)	C9—C10—H8	118.9
Ru1—N1—C5	114.6 (2)	N3—C11—H9	118.8
C1—N1—C5	119.4 (2)	C12—C11—H9	118.7
Ru1—N2—C6	115.2 (2)	C11—C12—H10	120.5
Ru1—N2—C10	125.6 (2)	C13—C12—H10	120.5
C6—N2—C10	118.9 (2)	C12—C13—H11	120.3
Ru1—N3—C11	126.0 (2)	C14—C13—H11	120.3
Ru1—N3—C15	115.3 (2)	C13—C14—H12	120.3
C11—N3—C15	118.7 (2)	C15—C14—H12	120.3
Ru1—N4—C16	114.4 (2)	C16—C17—H13	120.1
Ru1—N4—C20	126.7 (2)	C18—C17—H13	120.2
C16—N4—C20	118.9 (2)	C17—C18—H14	120.3
N1—C1—C2	122.3 (3)	C19—C18—H14	120.3
C1—C2—C3	119.1 (3)	C18—C19—H15	121.1
C2—C3—C4	119.3 (3)	C20—C19—H15	121.1
C3—C4—C5	119.3 (3)	N4—C20—H16	118.3
N1—C5—C4	120.7 (3)	C19—C20—H16	118.3
N1—C5—C6	115.5 (2)		
			
Cl1—Ru1—N2—C6	176.6 (2)	C22—Ru1—N3—C11	92.0 (7)
Cl1—Ru1—N2—C10	3.3 (2)	C22—Ru1—N3—C15	−87.8 (6)
Cl1—Ru1—N3—C11	88.8 (2)	N4—Ru1—C22—Cl1	−137 (12)
Cl1—Ru1—N3—C15	−91.0 (2)	N4—Ru1—C22—O2	−180 (8)
N3—Ru1—Cl1—O2	−150 (3)	C22—Ru1—N4—C16	88.3 (8)
N3—Ru1—Cl1—C22	121 (12)	C22—Ru1—N4—C20	−93.7 (8)
Cl1—Ru1—N4—C16	85.7 (2)	Ru1—N1—C1—C2	177.8 (2)
Cl1—Ru1—N4—C20	−96.2 (2)	Ru1—N1—C5—C4	−177.4 (2)
N4—Ru1—Cl1—O2	131 (3)	Ru1—N1—C5—C6	2.2 (3)
C21—Ru1—Cl1—O2	−52 (3)	C1—N1—C5—C4	1.5 (4)
Cl2—Ru1—N1—C1	−92.9 (3)	C1—N1—C5—C6	−178.9 (3)
Cl2—Ru1—N1—C5	85.9 (2)	C5—N1—C1—C2	−1.0 (5)
N1—Ru1—Cl2—O1	76 (3)	Ru1—N2—C6—C5	4.6 (3)
N1—Ru1—Cl2—C21	61 (3)	Ru1—N2—C6—C7	−174.0 (2)
Cl2—Ru1—N2—C6	−96.6 (2)	Ru1—N2—C10—C9	172.8 (2)
Cl2—Ru1—N2—C10	90.0 (3)	C6—N2—C10—C9	−0.3 (5)
N2—Ru1—Cl2—O1	155 (3)	C10—N2—C6—C5	178.5 (3)
N2—Ru1—Cl2—C21	139 (3)	C10—N2—C6—C7	−0.2 (4)
Cl2—Ru1—N3—C11	2.3 (3)	Ru1—N3—C11—C12	−179.3 (2)
Cl2—Ru1—N3—C15	−177.5 (2)	Ru1—N3—C15—C14	179.6 (2)
N3—Ru1—Cl2—O1	−21 (3)	Ru1—N3—C15—C16	−1.0 (3)
N3—Ru1—Cl2—C21	−37 (3)	C11—N3—C15—C14	−0.2 (4)
Cl2—Ru1—C22—O2	2 (5)	C11—N3—C15—C16	179.2 (2)
N1—Ru1—N2—C6	−2.7 (2)	C15—N3—C11—C12	0.5 (5)
N1—Ru1—N2—C10	−176.0 (2)	Ru1—N4—C16—C15	0.6 (3)
N2—Ru1—N1—C1	−178.6 (2)	Ru1—N4—C16—C17	179.7 (2)
N2—Ru1—N1—C5	0.2 (2)	Ru1—N4—C20—C19	−177.9 (2)
N1—Ru1—N3—C11	−92.4 (2)	C16—N4—C20—C19	0.1 (3)
N1—Ru1—N3—C15	87.8 (2)	C20—N4—C16—C15	−177.6 (2)
N3—Ru1—N1—C1	10.4 (2)	C20—N4—C16—C17	1.5 (4)
N3—Ru1—N1—C5	−170.8 (2)	N1—C1—C2—C3	0.2 (4)
N1—Ru1—N4—C16	−97.8 (2)	C1—C2—C3—C4	0.1 (4)
N1—Ru1—N4—C20	80.3 (2)	C2—C3—C4—C5	0.4 (5)
N4—Ru1—N1—C1	88.9 (2)	C3—C4—C5—N1	−1.2 (5)
N4—Ru1—N1—C5	−92.3 (2)	C3—C4—C5—C6	179.3 (3)
C21—Ru1—N1—C1	−87.9 (3)	N1—C5—C6—N2	−4.5 (4)
C21—Ru1—N1—C5	90.9 (3)	N1—C5—C6—C7	174.1 (3)
N2—Ru1—N3—C11	−155.0 (5)	C4—C5—C6—N2	175.1 (3)
N2—Ru1—N3—C15	25.2 (7)	C4—C5—C6—C7	−6.3 (5)
N3—Ru1—N2—C6	61.3 (6)	N2—C6—C7—C8	1.5 (5)
N3—Ru1—N2—C10	−112.1 (5)	C5—C6—C7—C8	−177.0 (3)
N2—Ru1—N4—C16	−176.8 (2)	C6—C7—C8—C9	−2.4 (4)
N2—Ru1—N4—C20	1.3 (2)	C7—C8—C9—C10	2.0 (5)
N4—Ru1—N2—C6	85.0 (2)	C8—C9—C10—N2	−0.6 (5)
N4—Ru1—N2—C10	−88.4 (2)	N3—C11—C12—C13	−0.5 (5)
N2—Ru1—C21—Cl2	−41 (3)	C11—C12—C13—C14	0.2 (4)
C21—Ru1—N2—C6	−92.9 (3)	C12—C13—C14—C15	0.1 (3)
C21—Ru1—N2—C10	93.7 (3)	C13—C14—C15—N3	−0.1 (3)
N2—Ru1—C22—O2	89 (9)	C13—C14—C15—C16	−179.4 (3)
C22—Ru1—N2—C6	173.7 (7)	N3—C15—C16—N4	0.3 (4)
C22—Ru1—N2—C10	0.4 (7)	N3—C15—C16—C17	−178.8 (3)
N3—Ru1—N4—C16	−0.9 (2)	C14—C15—C16—N4	179.6 (3)
N3—Ru1—N4—C20	177.2 (2)	C14—C15—C16—C17	0.5 (5)
N4—Ru1—N3—C11	−179.2 (2)	N4—C16—C17—C18	−1.6 (5)
N4—Ru1—N3—C15	1.0 (2)	C15—C16—C17—C18	177.4 (3)
C21—Ru1—N3—C11	−1.1 (3)	C16—C17—C18—C19	0.1 (4)
C21—Ru1—N3—C15	179.1 (3)	C17—C18—C19—C20	1.4 (5)
N3—Ru1—C22—O2	−101 (9)	C18—C19—C20—N4	−1.6 (5)

### Hydrogen-bond geometry (Å, º)

**Table:**

*D*—H···*A*	*D*—H	H···*A*	*D*···*A*	*D*—H···*A*
C3—H3···F2^i^	0.95	2.46	3.166 (4)	131
C4—H4···F1^ii^	0.95	2.41	3.257 (4)	148
C7—H5···F1^ii^	0.95	2.54	3.431 (4)	156
C8—H6···F2^iii^	0.95	2.50	3.265 (4)	138
C13—H11···F5^iv^	0.95	2.39	3.331 (4)	168

Symmetry codes: (i) −*x*+1, *y*+1/2, −*z*+1/2; (ii) −*x*+1/2, −*y*+1, *z*−1/2; (iii) *x*−1/2, −*y*+1/2, −*z*; (iv) *x*−1/2, −*y*+1/2, −*z*+1.
